# MDMA for PTSD and beyond: a new paradigm brings hope

**DOI:** 10.3389/fnhum.2024.1475013

**Published:** 2024-12-11

**Authors:** Scott Shannon, Jamarie Geller

**Affiliations:** ^1^Department of Psychiatry, University of Colorado, Denver, CO, United States; ^2^Wholeness Center, Fort Collins, CO, United States; ^3^Department of Psychiatry, University of Michigan, Ann Arbor, MI, United States

**Keywords:** 3, 4- methylenedioxymethamphetamine (MDMA), psychedelic-assisted psychotherapy, psychedelic, Post-traumatic Stress Disorder (PTSD), trauma, paradigm, psychotherapy, MDMA

## Introduction

We are experiencing a mental health crisis of unprecedented proportions. Over the last two decades this crisis grew from a perfect storm of rising insurance deductibles, escalating social stressors, the COVID-19 pandemic, and a critical workforce deficiency. Perhaps no mental health arena has a greater shortfall of effective treatment options than Post-traumatic Stress Disorder (PTSD) as the last new treatment was approved by the FDA 24 years ago (Davis and Hamner, 2024). Inadequate funding for research efforts and public resources available to expand services may in part stem from the inordinate prevalence of PTSD among women and disadvantaged populations. Women experience PTSD at two to three times the rate found in men (US Department of Veterans Affairs, 2023), and these rates are also significantly higher among African Americans, Latinos, Native Americans, and the homeless (Tortella-Feliu et al., 2019; Kwon et al., 2024; Armoon et al., 2024; Reyes et al., 2023). Neighborhood poverty also predicts the development of PTSD following a traumatic event (Ravi et al., 2023). Our trauma burden is unevenly distributed across society.

Trauma is unfortunately quite common. The National Center for PTSD estimates that over 13 million people in the US suffer from PTSD with 5% of our population diagnosed in any given year (US Department of Veterans Affairs, 2023). The annual cost in the US has been estimated at $232 billion (per person/year cost estimated at $19,630) in 2018 (Davis et al., 2022). Veterans as a group report high levels of trauma. According to the National Center for PTSD, 23% of veterans accessing the Veterans Administration services are diagnosed with PTSD (US Department of Veterans Affairs, 2023). Suicide, often linked to trauma and PTSD, has now become the leading cause of death for those serving in our Armed Forces (Kaplansky and Toussaint, 2024). For our society, the human suffering from trauma is immense and undeniable. Yet, our current treatments are inadequate (Smith et al., 2024).

Trauma-focused psychological therapies (TFPT) are the best-evidenced interventions for PTSD, and they are typically first line (Schrader and Ross, 2021). However, only about 50% of patients complete a course of treatment, and the response rates for those who do are roughly 50% (Schottenbauer et al., 2008). TFPTs are often not used by clinicians (Kline et al., 2021) due to fear of retraumatizing, dislike of manualized, rigid therapies and concern over symptom exacerbation (Finch et al., 2020). Even when high-quality TFPT is offered, patients often refuse to even consider this option because facing trauma in this way is extremely painful (Schottenbauer et al., 2008). Our indicated medications, such as sertraline and paroxetine which were first approved in the early 1990s, are minimally effective as evidenced by small effect size (0.28 and 0.23, respectively) (Hoskins et al., 2015, 2021) and often burdened with side effects. The need for innovation in PTSD care feels critical.

## MDMA and the psychedelic framework

MDMA represents our best hope for innovation in the treatment of PTSD in over a generation. The current application to the FDA for MDMA-assisted psychotherapy (MDMA-AP) is the first time that the FDA has considered this linkage of psychotherapy to a medication (DeBattista and Schatzberg, 2024). This combination serves as the core of the psychedelic framework. This framework has three components: container, catalyst and carrier (Shannon, 2023). The container is the psychotherapeutic model of support, safety and rapport as well as attention to set and setting. This creates the atmosphere of trust that is critical to work with traumatized patients. The catalyst is the psychedelic medication itself. This agent alters consciousness and the flow of information within it. This improves access to memories and feelings including traumatic material. Mechanistically, MDMA, psilocybin, and ketamine are all quite different, and their distinctive neurochemical properties continue to be elaborated. However, they all ultimately induce an altered awareness and enhanced processing of contents held in the psyche.

Finally, there is the carrier. This includes the use of curated music delivered by headphones, and eyeshades designed to drive the experience inward for therapeutic benefit. Without the carrier, the experience may be externally focused, and of little consequence other than amusement or distraction. While music is commonly considered a part of set and setting, in the psychedelic framework of psychotherapy it can also be considered a tool (along with eye shades and headphones) designed to support the inward focus of attention. Music does contribute to context, but it also plays a larger role in the inward nature of the experience by reducing cognitive hierarchy. This synergistic effect with psychedelics allows for greater flexibility in thought and perception characteristic of insight (Lebedev et al., 2016).

Together, these three basic components comprise psychedelic psychotherapy. This combination of container, catalyst and carrier ties the two disparate arms of conventional mental health treatment together for the first time. Both psychopharmacology and psychotherapy finally share unified goals and a common process. Thus, MDMA-assisted psychotherapy represents groundbreaking innovation and a true paradigm shift for mental health care (Zaretsky et al., 2024).

## MDMA and psychotherapy

Psychotherapy has evolved enormously since Sigmund Freud popularized psychoanalysis, but at its core it remains a talk therapy. Transcranial Magnetic Stimulation (TMS) is a more modern and less invasive variation on the shock therapies such as Electroconvulsive Therapy (ECT) that was introduced in the 1930s. Psychopharmacology, introduced in the 1950s, was the last true paradigm shift in mental health care. While the specific medications prescribed continue to evolve, the basic format is much the same: daily dosing of agents to reduce symptom load.

The application of specific psychedelic catalysts to durably improve psychiatric disorders through the alteration of consciousness when linked to psychotherapy is innovative at its core. It is a generally accepted principle that the magnitude of the success in psychotherapy flows from the quality of the relationship with the therapist. When there is a history of trauma, creating a container of trust and a context of safety becomes even more imperative. MDMA supports this therapeutic alignment process well by modulating a range of physiological effects. These include an increase in available monoamines accompanied by significant oxytocin release. This appears to occasion a sense of deep safety: a relaxed, more compassionate and prosocial, and less fearful state. With this level of emotional safety, trauma may be more easily accessed and reconsolidated (Feduccia and Mithoefer, 2018; Molla et al., 2023).

There are also network changes induced by MDMA that are implicated in the empathogenic effects, modulation of attention and processing of emotional and sensory information, and fear extinction (Gamma et al., 2000; Yu et al., 2024). A study carried out by Singleton et al. (2023) exploring brain activity and connectivity post-MDMA-AP for PTSD found an increase in resting-state amygdala-hippocampal connectivity, in addition to other shifts. MDMA induces a time limited enhancement of neuroplasticity (Ly et al., 2018), which may enhance this extinction learning and trauma-related reappraisal both during and after the dosing session. This process allow the body to digest trauma and move on without the rigid sequelae of PTSD.

Perhaps most importantly, MDMA facilitates something that often takes years to develop (and for some, never does): a decrease in defensiveness/avoidance. Theoretically, this allows the learning necessary for true healing to take place. MDMA has the potential to accelerate most forms of psychotherapy given the deep psychological support that it offers. The psychedelic model challenges the Western idea of a treatment as driven by external expertise and intervention, rather than internal wisdom and the innate capacity to heal. However, we would argue that for many modes of psychotherapy, as well as the majority of the psychiatric medications currently approved for use in the US, the mechanism remains mysterious. We must remain humble as we face the complexity of the human brain and consciousness. The Phase III studies published recently on MDMA-AP for PTSD demonstrate significant effect sizes and no evidence of serious adverse effects (Mitchell et al., 2021, 2023). Concerns over functional unblinding are part of the challenge we face in psychedelic medicine as a whole and not specific to MDMA (Goodwin et al., 2023).

Our current assessment tools, such as the Diagnostic and Statistical Manual (DSM) exhibit disappointing low rates of interrater reliability in actual practice. They also create a formidable challenge to our desire to move to a brain-based science as the diagnostic categories are often over inclusive and typically based on superficial phenomenology. In our opinion, the paradigm shift created by psychedelic medicine also calls for new methods of assessment that honor the centrality of the psyche and personal experience. Given the limited exposure to MDMA in this model (three doses) compared to conventional daily dosing, often with multiple pharmaceuticals, the biochemical exposure is considerably reduced.

MDMA treatment does have some risks, but they are manageable. In the Phase III studies these were almost exclusively time-limited responses during the day of ingestion: muscle tightness, nausea and the loss of appetite. Given the benefit of more durable improvement combined with a large effect size of 0.91, the potential benefits likely far outweigh the risks. Furthermore, this new paradigm also emphasizes the power of each individual to heal and it thus carries an optimism all too lacking in modern psychiatry with its emphasis on pathology.

## The potential of MDMA

To fully grasp the potential of MDMA in mental health care, we must first outline the therapeutic process. This agent confers psychotherapeutic benefit via a range of observed effects including the relaxation of defenses, an enhanced sense of safety, and the augmentation of compassion for oneself and others. This process allows for real insight and that often carries a reevaluation of one's values, behaviors, and core beliefs. In our perspective, this shift in awareness has the potential to span domains of psychopathology beyond trauma. If one can learn to regulate their nervous system in the context of this deep trauma work, it also allows for increased tolerability of psychotherapeutic work on other clinical issues, both during and after a dosing session.

It is hard to assess the promise of MDMA as it carries transdiagnostic capacity. Clearly, MDMA carries broad potential for many of the facets of human trauma, including developmental, attachment, and repeated traumas. MDMA also has potential utility for several other diagnostic categories, especially those which may have roots in trauma (addiction, anxiety, depression, and personality difficulties) (Shannon et al., 2021). However, it could also be quite useful in eating disorders where self-compassion and acceptance may be hard won.

When MDMA-assisted therapy is approved for use in adults, sponsors will be compelled to investigate safety and effectiveness in traumatized adolescents and children as well (Kangaslampi and Zijlmans, 2023). We believe this is another group that deserves access to a treatment that has the potential to completely change the trajectory of their lives. Taking this reasoning a step further, perhaps traumatized parents could be treated prior to any generational carryover of their untreated burden (Narayan et al., 2021). Furthermore, given the pernicious capacity of trauma, as defined by the Adverse Childhood Experiences (ACEs) literature (Gilgoff et al., 2020), this work has the potential to dramatically reduce the burden of pain, suffering and illness carried through a lifetime.

Given its relational and empathogenic effects, MDMA-assisted therapy could speed work with couples, families, and groups. We see this as an invaluable tool in fractured groups and communities, who could use an infusion of trust and the facilitation of clear, compassionate communication.

Additionally, MDMA may have a place with various demographic groups, especially those who have been disenfranchised and for whom access to meaningful psychiatric care has historically been limited. Often, these groups have faced enormous pain due to structural factors and deserve access to this potentially profound healing modality. We encourage ongoing investigation into the potential use of MDMA in veterans, with additional focus on the justice-involved, people of color, and LGBTQ communities. Does the effective treatment of trauma carry a social justice agenda? MDMA appears to enhance our awareness and engagement in a range of broader issues around our shared humanity and interconnection. In this manner, the broader application of MDMA might drive changes not only to the treatment of patients, but to care delivery system itself.

## Discussion

At this juncture, to both understand and effectively evaluate the transformative potential of this novel treatment modality, we need to reimagine the fabric of mental healthcare in this country. Over the last three decades the insurance industry has increasingly controlled the delivery of mental health care in the US with an emphasis on rapid symptomatic management (Godman et al., 2018). This has also frustrated attempts to develop and implement parity provisions for mental health (Langeland and Olaff, 2022). Prevention has been for the most part ignored in on-going care delivery (Kamra et al., 2024). We believe this shift to the specific psychotherapeutic paradigm that underlies psychedelic medicine will create more context for intensive treatment, prevention and much needed therapeutic innovation. By emphasizing personal experience and the power of the psyche to heal, we also bring more optimism and attention to the humanistic needs often lacking in our current treatment approach.

A reimagining of the centrality of trauma could also improve access to and acceptance of programs that are more preventative in nature, and that may help mitigate the vast sequelae of trauma sooner. Can psychiatry also demand complex and potentially expensive treatments not unlike a hip replacement or the placement of a stent? Economic evaluation outlines that effective treatment of severe PTSD can save more than $100,000 per case (Davis et al., 2022). The consequences of trauma and the linked functional impairment drive much of the cost across our entire healthcare system (Davis et al., 2022). The positive trials assessing MDMA-AP not only represent the success of a novel type of treatment, but it also demands a reevaluation of what mental healthcare is, and what it can be.
